# Co-circularity opponency in visual texture

**DOI:** 10.1038/s41598-018-38029-w

**Published:** 2019-02-04

**Authors:** Hiromi Sato, Frederick A. A. Kingdom, Isamu Motoyoshi

**Affiliations:** 1 Department of Informatics, Kogakuin University, Tokyo, Japan; 2 McGill Vision Research, Department of Ophthalmology, McGill University, Québec, Canada; 3 JSPS Research Fellow, Tokyo, Japan; 4 Department of Life Sciences, The University of Tokyo, Tokyo, Japan

## Abstract

It is well known that the human visual system is sensitive to co-circularity among oriented edges, which are ubiquitous features of object contours. Here, we report a novel aftereffect in which the appearance of a texture is dramatically altered after adaptation to a texture composed of elements with co-circular structure. Following prolonged viewing of a texture made of pairs of adjacent Gabor elements arranged to form obtuse angle co-circular pairs, i.e. shallow curves, a subsequently viewed random texture appears to be composed of acute angle, i.e. near-parallel pairs. Conversely, adaptation to a texture made of parallel pairs causes a random texture to appear to be composed of shallow curves. This suggests that mechanisms sensitive to co-circularity are organized in an opponent manner, with one pole sensitive to shallow curves the other parallel shapes. This notion was tested further in a non-adaptation experiment in which co-circular and non-co-circular Gabor pairs were mixed within a single texture. Results revealed summation between pairs that fell on one side of the opponent continuum, and cancellation between pairs that fell on opposite sides of the continuum. Taken together these results support opponent interactions between mechanisms sensitive to pairwise co-circular texture features.

## Introduction

In natural scenes, contours often contain regions that are “co-circular”^1^, that is they have a constant radius of curvature or constant second derivative of orientation^2^. Examples of co-circular structure are straight lines, smooth curves, corners and acute angles. If a contour comprises discreet line fragments, it is said to be co-circular when the fragments lie tangentially on a common circle^2^. Co-circular structure is particularly ubiquitous in the outline shapes of physically coherent objects (apples, faces, pebbles) and in the markings of textured surfaces (hair, grass, tree bark). While a special case of co-circularity, collinearity, has attracted considerable attention in both the psychophysical^3–9^ and neurophysiological^10–12^ vision literature, the role of co-circular structure in vision and the mechanisms that encode it have so far attracted relatively little attention^13–17^.

To investigate the role of co-circular structure in texture perception, Motoyoshi and Kingdom^15^ created textures composed of random arrays of element-pairs (elements were small oriented Gabor patches) each defined by one of a variety of orientation and positional relationships, of which a sub-set were co-circular arrangements. They measured the ability of human observers to discriminate textures whose Gabor pairs had a specific orientation/positional arrangement from textures whose pairs had a random orientation/positional relationship, and found that textures made from co-circular element-pairs were the most easily discriminable. This finding is in keeping with the more general finding from studies of synthesized natural textures that spatial relationships between local orientation signals play an important role in the perception of natural images^18–20^.

**Figure 1:**
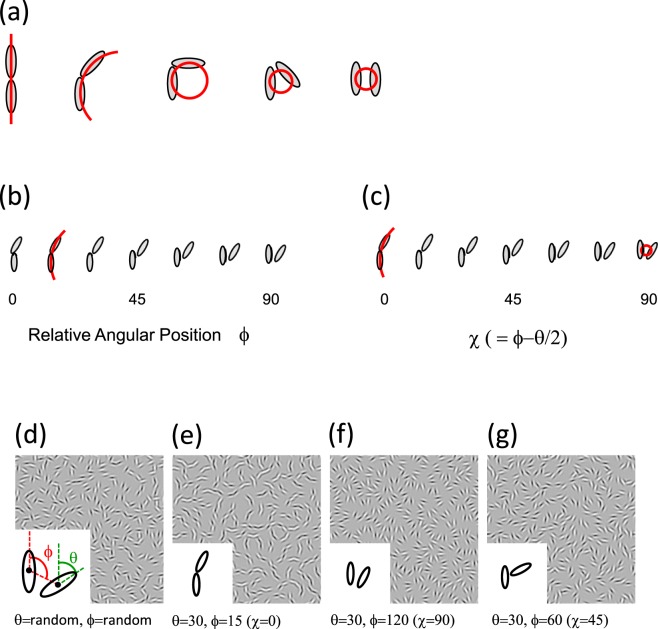
(**a**) Pairs in co-circular relationships, with orientation differences θ, as defined in the inset bottom left, from left to right: 0, 45, 90, 45 and 0 deg, termed here collinear, obtuse-angle, right-angle, acute-angle, parallel. (**b**) The pairs with orientation difference 30 deg are placed in a space of φ. (**c**) The pairs are placed in the transformed space of χ defined as φ - θ/2; when χ = 0 deg (obtuse-angle) and 90 deg (acute-angle) they are co-circular. (**d**–**g**) example textures made from Gabor pairs with an orientation difference θ of 30 deg and various angular positions φ (see inset); (**d**) random orientation/position pairs; (**e**) obtuse-angle co-circular pairs; (**f**) acute-angle co-circular pairs; (**g**) angular position of 60 deg.

Figure 1 shows examples of the stimuli employed by Motoyoshi and Kingdom^15^, also used here. The textures in Fig. 1d–g are made from arrays of Gabor element pairs, individual examples of which are illustrated in Fig. 1b,c. The Gabor pairs in Fig. 1a are all co-circular, and range from collinear (straight), obtuse-angle (smooth curve), right-angle, acute-angle (sharp curve) to parallel. Figure 1d–g show a series of textures, two of which are made from co-circular Gabor pairs, specifically Fig. 1e from obtuse-angle and Fig. 1f from acute-angle co-circular pairs. For comparison Fig. 1d shows a texture made from pairs with random orientation/positional relations and Fig. 1g. pairs with a particular orientation/positional relationship that is non-co-circular. The figure illustrates how easily one can distinguish co-circular from random as well as non-co-circular Gabor-pair textures.

The Gabor pairs in Fig. 1 are defined by a specific orientation difference θ and a specific relative angular position φ, as illustrated in the inset at the bottom left of Fig. 1d. In Fig. 1b the pairs are arranged in terms of relative angular position. When φ = θ/2 or φ = θ/2 + 90, the pairs are in a co-circular relationship, that is they are tangent to (φ = θ/2), or radial along (φ = θ/2 + 90), a common circle. In Fig. 1c the space of element pairs is transformed into a dimension termed χ, defined as φ = θ/2. This dimension produces a continuum in which the extreme points of χ, 0 and 90 deg, define pairs that are co-circular (Fig. 1c). Specifically when χ is 0 deg, the pair is obtuse-angle co-circular (the left in Fig. 1b and second from the left in Fig. 1c) and when χ is 90 deg the pair is acute-angle co-circular (the right in Fig. 1b and third from the left in Fig. 1c).

Figure 1e,f illustrate how obtuse-angle and acute-angle co-circular textures elicit different percepts. In this communication we consider whether these different percepts reflect an opponent representation of co-circular structure, analogous to the red-green and blue-yellow opponencies in color vision^21^. In one experiment we adapted subjects to a texture with an obtuse-angle co-circular (Fig. 1e) structure and found that this made a random texture (Fig. 1d) appear predominantly acute-angle co-circular. In a subsequent experiment we found that mixtures of acute- and obtuse- angle co-circular structures cancel perceptually. Our results support the idea that co-circularity is represented as an opponent dimension of vision.

## General Methods

### Subjects

One naïve observer (S2), and two of the authors (S1, S3), all with normal or corrected-to-normal vision participated in the experiments. These three observers were the only persons tested, i.e. no one was rejected because she/he was unable to perform the task or for any other reason^22^. All the experiments followed the Declaration of Helsinki guidelines, and were approved by the ethics committee for experiments on humans at Graduate School of Arts and Sciences, The University of Tokyo with completed consent forms. All observers provided informed consent.

### Apparatus

Visual stimuli were generated by a graphics card and displayed on a 27-inch LCD monitor (BENQ XL2735) with a refresh rate of 60 Hz. From a viewing distance of 57 cm, the LCD’s pixel resolution was 1.41 min/pixel and mean luminance was 56 cd/m^2^. The resolution of pixel value was 8 bit. The luminance of the LCD monitor was calibrated using a spot photometer (model S370, United Detector Technology).

### Stimuli

**Figure 2:**
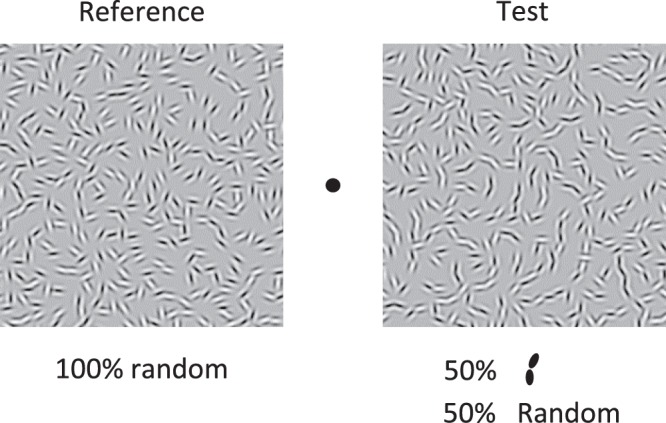
Example stimuli used in Experiment 1. The test stimuli were composed of both signal and noise pairs. The χ of the signal pairs were 0, 15, 30, 45, 60, 75 or 90 deg. The proportion of signal pairs was 13, 25, 50 or 100%, with the remainder noise pairs. In the example test stimulus shown here, the χ of the signal pairs was 0 deg and the proportion of signal pairs was 50%.

The stimulus was a square texture field of 7.0 deg on a side that consisted of arrays of pairs of Gabor micropatterns (Fig. 2). Each Gabor pattern was a sinusoidal grating with a spatial frequency of 7.1 c/deg windowed by a Gaussian with a standard deviation of 0.42 deg. It had a luminance contrast of 1.0 and random spatial phase. Each Gabor in a pair was placed with a centroid-to-centroid separation of 0.21 deg. The pairs were randomly placed within the texture field under the constraint that the center-to-center separation between adjacent pairs was a minimum of 0.42 deg. Therefore, the elements or element pairs never overlapped with each other. The absolute orientations of the Gabor pairs in all experiments were randomized for every stimulus presentation.

### Procedure

During testing there were always two stimuli, a test and comparison stimulus, presented 4.7 deg either side (centre-to-centre) of a 0.09 × 0.09 deg fixation dot on a homogeneous grey background. Stimuli were presented for 333 ms unless otherwise stated. The location (right or left) of the test stimulus was counter-balanced across trials. On each trial, observers were asked to indicate by button press which texture appeared to contain more obtuse-angle curves. No feedback was given.

## Experiment 1: Sensitivity to Co-Circular Structure

The first experiment measured the sensitivity of our observers to co-circularity in our textures. The orientation difference θ of the signal pairs was fixed at 30 deg. For the signal pairs in the test stimulus there were 7 levels of angular position φ: 15, 30, 45, 60, 75, 90, 105 resulting in χ values of 0, 15, 30, 45, 60, 75, 90. For the noise pairs in both test and comparison, both θ and χ were randomly selected from 0-90. The proportions of signal pairs in the test were 13, 25, 50 or 100%, with the remainder noise pairs. Thus there were 28 conditions: 7 values of χ × 4 signal-pair proportions.

The task on each trial was always to choose the texture that appeared to have more obtuse-angle curvature. Within a single session, all 28 conditions were interleaved and presented in random order. The session terminated when the number of trials in all conditions exceeded 10. The sessions were repeated several times, until at least 50 trials were conducted for each condition.

### Results

**Figure 3:**
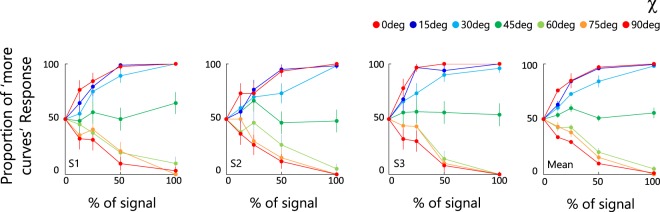
Results from Experiment 1. Each graph shows the proportion of test stimuli perceived as more obtuse-angle as a function of the proportion of signal pairs in the test stimuli, with each curve a different value of χ. Different graphs are for different observers, with the right-most panel the mean across observers. Error bars represent ± 1 SEM.

Figure 3 shows the proportion of responses in which observers responded that the test stimulus contained more obtuse-angle curvature, as a function of the proportion of signals pairs for each value of χ. Individual observer panels are shown on the left, the average of all observers on the right. Each color represents a different χ, with the exception that the two co-circular extremes are both red. Points greater than 50% indicate that the observers chose the test, and points less than 50% that the observers chose the comparison, as containing more obtuse-angle curvature. The two-way repeated-measure ANOVA showed significant main effects of both % of signal (F(3, 56) = 7.27, p < 0.001) and of χ (F(6, 56) = 382.46, p < 0.001), and a significant interactions between the two (F(18, 56) = 26.34, p < 0.001).

When χ was 0 deg (obtuse-angle co-circular), the proportion of responses always exceeded 50% and reached 100% once the signal proportion exceeded about 50%. On the other hand when χ was 90 deg (acute-angle co-circular), the proportion of response was always less than 50% and declined to 0% once the signal proportion reached 100%. Intermediate levels of χ produced curves in between the two co-circular extrema. At a χ of 45 deg, the proportion of responses remained more-or-less constant at 50% for all signal proportions, indicating that observers perceived those stimuli as having the same curvature structure as the comparison stimuli, even when the test had 100% signal. These results show that observers were sensitive to the obtuse-angle and acute-angle structure in the textures as well as to the degree of χ.

## Experiment 2: Co-Circularity Aftereffect

Having confirmed that observers can readily discriminate co-circular from random textures and are sensitive to the degree of χ, we now test whether prior adaptation to textures with on the one hand obtuse-angle and on the other acute-angle co-circular structure alters the perception of subsequently presented textures.

**Figure 4:**
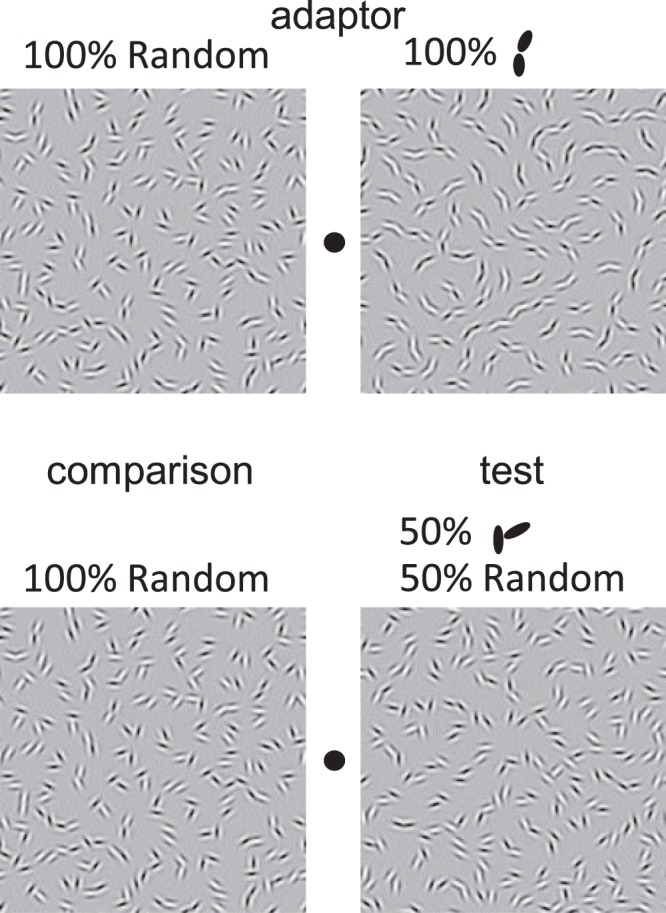
Example stimuli used in Experiment 2. The adaptation stimuli (top) were either ‘noise’ (left), containing 100% random or ‘signal’ (right) containing 100% of Gabor pairs with χ’s either 0, 45 or 90 deg (0 deg in this example). In the test phase (bottom) the comparison and test textures were presented on the sides corresponding to the noise and signal adaptors respectively. The χ of the test stimulus was varied from 0 to 90 deg in 15 deg step. The proportion of signal in the test stimulus was always 50**%**.

During the adaptation phase two adaptor textures were presented. One comprised only signal pairs – the ‘signal adaptor’ - and was displayed on the side of fixation where the test stimulus was later presented. The other was a noise texture – the ‘noise adaptor’ (Fig. 4). There were 3 conditions for the signal adaptor: χs of 0, 45, or 90 deg. The test and comparison stimuli were similar to those in Exp. 1, i.e. all signal element pairs had an orientation difference of 30 deg, with the test comprising both signal and noise pairs (the former with χs varying in 15 deg steps resulting in 7 different pairs), and the comparison pairs with random φ (Fig. 4). The test stimulus always contained 50% signal pairs. There was a total of 21 conditions: 7 levels of the test χ × 3 levels of the adaptor χ.

Observers initially viewed the adapting textures using central fixation for 5 sec. On each trial, following a top-up adaptation period of 5 sec (10 stimulus refreshes) interspersed with a blank field of 666 ms, test and comparison textures were presented for 833 ms. In this experiment, we employed a longer test presentation duration than in Experiment 1 in order to help observers distinguish more easily the test and adaptor stimuli. The observers judged which texture appeared to contain more obtuse-angle curves. No feedback was given. Within a single session, the 7 levels of χ of signal A were interleaved, and each condition was randomly presented. The location of the adaptors was counter-balanced across sessions. Since the signal adaptor was always presented on the same side as the test stimulus, the observer tended to select the same side. To avoid this bias, we manipulated the number of trials for each condition within one session. That is, the number of trials for the condition in which the observer chose the reference or test stimulus was reduced. Although in the condition with the smallest number of trials (obtuse-angle signal adaptor followed by acute-angle test, and acute-angle signal adaptor followed by obtuse-angle test) the number of trials was 8, the standard errors were nevertheless zero for all observers. The session terminated when the number of trials in each condition became 2–8. Sessions were repeated several times, until at least 8 trials of the data had been collected for each condition.

### Results

**Figure 5:**
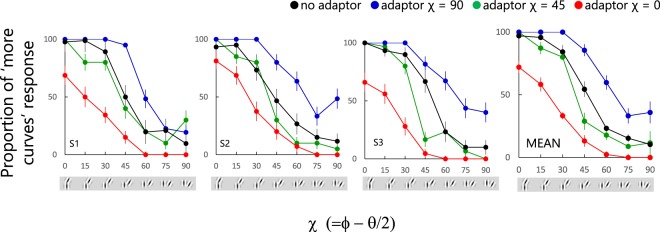
Results from Experiment 2. Proportion of test stimuli perceived as having more obtuse-angle curves as a function of χ, with different curves for different adaptor χs, for three observers. The black symbols show results from the 50% signal condition from Experiment 1, i.e. without adaptation. The right panel shows the mean across observers. Error bars represent ± 1 SEM.

Figure 5 shows the proportion of responses in which observers chose the test stimulus as containing more obtuse-angle curvature as a function of χ in both adaptor and test stimuli. The right panel shows the average of all observers, with the panels to the left the results for each observer. Each color of circles represents the results for each adaptation χ: blue circles for χ = 90 deg, green circles for χ = 45 deg, and red circles for χ = 0 deg. The black circles replot the data from the (no adaptation) Exp. 1 when the signal proportion was 50%. Points greater than 50% indicate that the observers tended to choose the test stimulus and points lower than 50% the comparison stimulus as the one with more obtuse-angle curves. The two-way repeated-measure ANOVA showed significant main effects of both χ (F(6, 56) = 244.19, p < 0.001) and of adaptation condition (F(3, 56) = 148.06, p < 0.001), and significant interactions between the two (F(18, 56) = 5.87, p < 0.001). Multiple comparison revealed that the responses for 0 deg 45 deg, and 90 deg adaptors were significantly different from the no-adaptation condition (t(56) = 12.52, p < 0.001; t(56) = 2.86, p = 0.006; t(56) = 8.34, p < 0.001, respectively).

For all observers, the proportion of test stimuli perceived as having more obtuse-angle curves was increased following adaptation to the acute-angle co-circular adaptors while reduced following adaptation to the obtuse-angle co-circular adaptors. This “co-circularity aftereffect” suggests an opponent interaction between the perception of obtuse- and acute- angle co-circular structure.

## Experiment 3: Summation and Cancellation Between Opposite Poles of Co-Circular Structure

**Figure 6:**
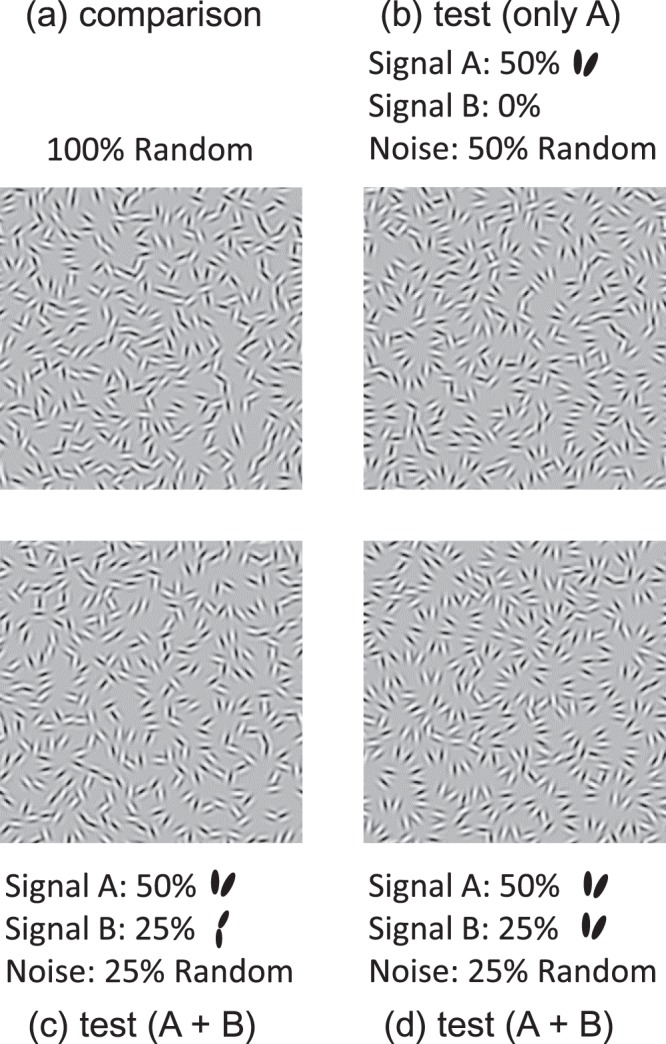
Example stimuli in Experiment 3. (**a**) A comparison stimulus composed of noise pairs with random φ. (**b**) An example test stimulus composed of 50% of signal A with χ of 90 deg and 50% of noise pairs with random φ (this condition was from Exp. 1 and not included in Exp. 3). (**c**) Test stimulus composed of 50% of signal A with χ of 90 deg, 25% of signal B with χ of 0 deg, and 25% of noise pairs with random φ. (**d**) Test stimulus composed of 50% of signal A with χ of 90 deg, 25% of signal B with χ of 90 deg, and 25% of noise pairs with random θ and  φ.

**Figure 7:**
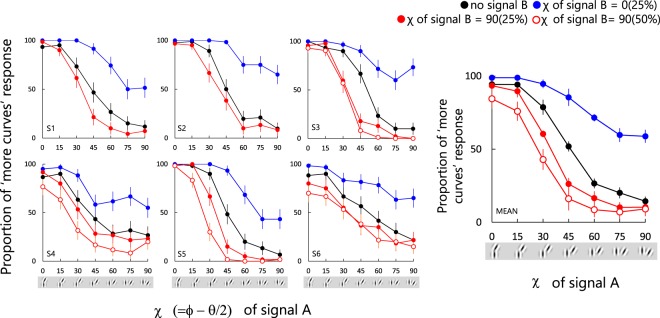
Results from Experiment 3. Each plot shows for each observer the proportion of times the test stimulus was chosen as containing more obtuse-angle pairs, as a function of signal A χ. Different curves are for different signal B conditions, as described in the legend. The black symbols plot the results from the Experiment 1, i.e with no signal B. The right-most panel show the average across observers. Error bars represent ± 1 SEM.

If obtuse- and acute-angle structures are represented in an opponent fashion, they should cancel when mixed together in a single texture. In this experiment, we measured performance for detecting obtuse-angle curvature in test stimuli composed of a mixture of signal pairs. We term the two types of signal pairs A and B; examples are shown in Fig. 6. The χ of signal A was varied in 15 deg steps from 0 to 90 deg, resulting in 7 different pairs. The proportion of signal A in the test stimulus was always 50%. The χ of signal B was either 0 (obtuse-angle) or 90 deg (acute-angle): when 0 deg, the proportion was 25%, when 90 deg the proportion was either 25% or 50%. The 0% pedestal condition in Fig. 6b was the same as one of the conditions in Experiment 1, so it was not included in this experiment: the results of this condition are shown in Fig. 7 as the black curve. The remaining pairs in the test stimulus were noise pairs, i.e. with random θ and  φ, and the comparison texture was composed of noise pairs. As with the previous experiments the orientation difference of all signal element pairs was 30 deg.

On each trial, the test stimulus was presented on one side of fixation and the comparison stimulus on the other. Stimulus exposure time was 333 ms. Observers were asked to indicate by button press which of the test or comparison stimulus appeared to contain more obtuse-angle curves. No feedback was given. The seven conditions of signal A χ were interleaved within a session, but each signal B χ was fixed within a session. A session terminated when the number of trials in all conditions exceeded 30. Sessions were repeated several times, until at least 60 trials were collected for each condition. After the experiment, the proportion of responses at which the test stimulus was chosen for each condition for each observer was obtained. Four naives (S2, S4–6) and 2 authors (S1, S3) participated in the experiment. The six observers were the only persons tested, and no one was rejected because she/he was unable to perform the task or for any other reason^22^.

### Results

Figure 7 shows the proportion of responses in which observers responded that the test stimulus contained more obtuse-angle curvature as a function of signal A χ. Panels show the results for each observer, with the right-most panel the average across observers (Note only 4 observers (S3-S6) participated in the 50% signal B condition). Blue circles represent the results when signal B χ was 0 deg and proportion 25%, red circles when signal B χ was 90 deg and proportion 25%, and red open circles when signal B χ was 90 deg and proportion 50%. The black circles replot the data from the 1st experiment where no signal B was added (Fig. 6b). Points greater than 50% indicate that the observers tended to choose the test stimulus, and points lower than 50% that the observers tended to choose the comparison stimuli. The two-way repeated-measure ANOVA showed significant main effects of both χ of signal A (F(2,105) = 200.61, p < 0.001) and of χ of signal B (F(6,105) = 201.12, p < 0.001), and significant interactions between the two (F(12,105) = 11.39, p < 0.001). Multiple comparison revealed that the responses for 0 deg and 90 deg signal A were significantly different from the no-signal A condition (t(105) = 13.96, p < 0.001; t(105) = 5.46, p < 0.001, respectively).

The results show that when the added signal B χ was 0 deg, the psychometric function shifted upward whereas when it was 90 deg it shifted downwards. This evidences perceptual cancellation between the obtuse-angle and acute-angle co-circular pairs. However the cancellation is asymmetric. Whereas addition of obtuse-angle (0 deg) signal B to acute-angle signal A made the stimuli appear random (rightmost data point on the blue curve), addition of acute-angle signal B (90 deg) to obtuse-angle signal A (leftmost points on the red curves) had comparatively little effect. This asymmetry mirrors the black curve (no signal B) which is biased towards obtuse-angle sensitivity (see also Motoyoshi and Kingdom)^15^. The degree of asymmetry however is observer-dependent, and in the case of S6 absent altogther.

## General Discussion

### Results Summary

Using textures composed of arrays of Gabor-pairs we considered whether obtuse- and acute-angle co-circular pair textures are perceived as opponent structures. Each Gabor-pair was defined by one of a variety of orientation and positional relationships, of which a sub-set were co-circular obtuse-angle or co-circular acute-angle curves. The results showed that (1) observers are sensitive to the obtuse-angle and acute-angle structures in the textures; (2) following adaptation to the acute-angle adaptors, subsequently viewed textures appeared to have more obtuse-angle curves, and vice versa; (3) when the obtuse-angle and the acute-angle co-circular pairs were mixed within a single texture, the texture was perceived to have neither type of structure. These results suggest that co-circularity is an important dimension in texture perception, and that obtuse- and acute- angle co-circularities are likely organized in an opponent manner, analogous to the opponency between red and green in color vision.

In the present study we only employed Gabor pairs with an orientation difference of 30 deg, a difference which Motoyoshi and Kingdom^15^ showed was close to the orientation difference with the highest sensitivity (45 deg). In pilot studies, we measured for one observer the shifts in psychometric functions from adaptation for orientation differences of 0 deg or 60 deg, using the same protocol as Exp. 2, and found similar results. Similar results to the cancellation Exp. 3 were also found for this same observer using 0 and 60 deg orientation-difference Gabor pairs. Although conditions with orientation differences other than 30 deg remain to be explored, we presume that the co-circularity opponency found in this study is a characteristic of any orientation difference, i.e. not limited to 30 deg.

### Orientation and curvature

The absolute orientations of the Gabor were randomized for every stimulus. Therefore, the orientation spectrum in every texture was broadband. In addition, in experiment 2, during the 5 sec adaptation period, the 0.5 sec adaptor stimulus was refreshed 10 times with a completely new set of random orientations. Thus, at any one location in the stimulus there was a random relationship between the Gabor orientations across adaptor presentations and a random relationship between the Gabor orientations between the adaptor and test. Therefore the aftereffect results cannot be attributable to well-known orientation interactions such as the tilt aftereffect^23^ or simultaneous orientation contrast^24^. Rather, the results are best attributable to interactions at higher levels of processing that combine the orientational and positional information of the two members of each Gabor pair. What, however, of the possible involvement of curvature processing? All signal Gabor-pairs in the present study shared the same orientation difference of 30 deg and varied only in their positional relationships. However, varying the positional relationships resulted in pairs that at the two extremes of χ formed obtuse-angle and acute-angle co-circular structures. It is known that adaptation to curvature can repulsively shift the perceived curvature of subsequently presented curves^25–27^. However the curvature aftereffect is strongly selective to the overall orientation of the curve^27^, so we would not expect strong curvature aftereffects in stimuli in which the overall orientation of each Gabor pair was randomized. Moreover one can only speculate as to the likely magnitude of any adaptation-induced shifts in the perceived curvature of our signal Gabor pairs given that they vary only in their positional relationship. Thus although we cannot rule out the possibility that conventional curvature aftereffects might contribute towards the results reported in our 2^nd^ Experiment, it seems unlikely.

### Spatial autocorrelation

Recent studies on texture perception, especially those using texture synthesis, have demonstrated the importance of the relationship between local orientation signals for the perception and discrimination of natural textures^18–20,28^. These findings are consistent with our results. However together with our previous data^15^, the present results suggest that the visual system is primarily sensitive to a specific relationship among orientation signals, i.e., co-circularity, and that it represents the relationship in an opponent manner, i.e., in terms of acute vs. obtuse angles. In turn this supports the idea that the visual system represents higher order orientation statistics via a compact code, in this case co-circularity with acute-obtuse polarity.

### Contours versus textures

Mounting evidence suggests that contours and textures are processed by different mechanisms (reviewed by Gheorghiu, Kingdom and Petkov)^29^. Part of this evidence concerns the putative role of neurons in the visual cortex that possess surrounds that nonlinearly inhibit the responses from their classical-receptive-field centres. Such neurons exhibit what has been termed “iso-orientation surround suppression”, or IOSS, meaning that the neuron’s response to an oriented line positioned within its classical receptive field is suppressed by similar orientations placed outside of it^30–35^. Models of such neurons have shown that they are sensitive to the contours but not textures in images of natural scenes, leading to their being labeled as “de-texturizers”^29^. Our textures composed of obtuse-angle pairs resemble an array of contours, whereas those composed of acute-angle pairs appear more texture-like. It is therefore possible that in our textures containing both acute and obtuse-angle pairs, the acute-angle pairs suppress the perception of the obtuse-angle pairs via IOSS (and see Kingdom and Prins)^36^. In other words the co-circularity opponency revealed in the present study might be partly mediated by IOSS. It will therefore be interesting to model the responses of IOSS neurons to our textures to determine how much of our results might be thus explained.

## Data Availability

The dataset is available online on figshare public repository.
